# Serum anti‑TSTD2 antibody as a biomarker for atherosclerosis‑induced ischemic stroke and chronic kidney disease

**DOI:** 10.3892/mi.2022.64

**Published:** 2022-12-21

**Authors:** Masaaki Kubota, Bo-Shi Zhang, Shu-Yang Li, Yoichi Yoshida, Hao Wang, Akihiko Adachi, Tomoo Matsutani, Seiichiro Mine, Toshio Machida, Ikuo Kamitsukasa, Takeshi Wada, Akiyo Aotsuka, Kenichiro Kitamura, Hirotaka Takizawa, Hideyuki Kuroda, Yasuo Iwadate, Takaki Hiwasa

**Affiliations:** 1 Department of Neurological Surgery, Graduate School of Medicine, Chiba University, Chiba 260-8670, Japan; 2 Comprehensive Stroke Center, Chiba University Hospital, Chiba 260-8677, Japan; 3 Department of Anesthesiology, Stroke Center, The First Affiliated Hospital and Health Science Center, Jinan University, Guangzhou, Guangdong 510630, P.R. China; 4 Department of Neurological Surgery, Chiba Prefectural Sawara Hospital, Chiba 287-0003, Japan; 5 Department of Neurological Surgery, Chiba Cerebral and Cardiovascular Center, Chiba 290-0512, Japan; 6 Department of Neurosurgery, Eastern Chiba Medical Center, Chiba 283-8686, Japan; 7 Department of Neurology, Chiba Rosai Hospital, Chiba 290-0003, Japan; 8 Department of Neurology, Chibaken Saiseikai Narashino Hospital, Chiba 275-8580, Japan; 9 Department of Internal Medicine, Chiba Aoba Municipal Hospital, Chiba 260-0852, Japan; 10 Kitakurihama Takuchi Clinic, Yokosuka, Kanagawa 239-0807, Japan; 11 Port Square Kashiwado Clinic, Kashiwado Memorial Foundation, Chiba 260-0025, Japan; 12 Medical Project Division, Research Development Center, Fujikura Kasei Co., Saitama 340-0203, Japan

**Keywords:** autoantibody, thiosulfate sulfurtransferase-like domain-containing 2, atherosclerosis, acute cerebral infarction, chronic kidney disease

## Abstract

Autoantibodies can be used in the early diagnosis and treatment of atherosclerosis-related diseases. Using ProtoArray^®^ screening of samples from patients with atherosclerosis, the present study identified thiosulfate sulfurtransferase-like domain-containing 2 (TSTD2) as a novel atherosclerosis antigen. The serum TSTD2 antibody levels were then quantified using an amplified luminescent proximity homogeneous assay-linked immunosorbent assay. This demonstrated the levels of TSTD2 antibodies (TSTD2-Abs) to be significantly higher in patients with acute cerebral infarction or chronic kidney disease than in healthy donors. The TSTD2-Ab levels were also found to be higher in males, older adults, smokers, in those who consumed alcohol regularly, and in those with hypertension. Furthermore, Spearman's rank correlation analysis revealed TSTD2-Ab levels to be strongly associated with measures of atherosclerosis severity, including plaque scores, intima-media thickness of the carotid artery and the cardio-ankle vascular index. Thus, TSTD2-Abs may thus be a promising novel biomarker for atherosclerosis-related cerebral infarction and kidney disease.

## Introduction

Ischemic stroke is one of the most common vascular disorders worldwide and, despite notable advancements being made in treatments and diagnostic imaging techniques, it is still associated with high mortality and morbidity rates (1,2). Chronic kidney disease (CKD) is also a serious public health concern with a high incidence rate (3). Atherosclerosis is the main cause of both ischemic stroke and CKD (4,5). The immune response to atherosclerosis causes the production of autoantibodies in the sera of patients (6). These can potentially be used as biomarkers for preventive diagnosis and for the identification of the disease type of atherosclerosis-related diseases. Recent studies have demonstrated that serum autoantibodies play a critical role in various diseases (7,8). In previous studies, the authors identified several autoantibodies associated with ischemic stroke, including RPA2, PDCD11, ATP2B4, BMP-1, MMP1, CBX1, DNAJC2, AP3D1, DIDO1 and SERPINE 1 (9-15). These may all be applied to diagnosis and treatment. The present study examines the use of the anti-thiosulfate sulfurtransferase-like domain-containing 2 (TSTD2) autoantibody as a novel biomarker for atherosclerosis-related cerebral infarction and CKD in the sera of patients with atherosclerosis.

## Materials and methods

### Patient samples

Serum samples were obtained from patients who suffered an ischemic stroke and from healthy donors (HDs). The present study analyzed 684 serum samples, including 275 from patients with acute ischemic stroke (AIS), 300 from patients with CKD and 109 from HDs. The AIS group consisted of 196 patients with acute cerebral infarction (aCI) and 79 patients with transient ischemic attack (TIA). The serum samples of the patients with aCI and TIA were collected at Chiba Prefectural Sawara Hospital, Chiba Rosai Hospital, and Chiba Aoba Municipal Hospital. The aCI samples were collected within 2 weeks of the diagnosis of atherothrombotic brain infarction. The median (range) of age in years of the HD, aCI and TIA subjects were 60 (45-90), 77 (58-85) and 73 (26-90), respectively. The subject information of the Sawara stroke cohort is summarized in Table SI.

The CKD serum samples were obtained from the Kumamoto cohort (16,17), which included 145 samples from patients with type-1 (diabetic kidney disease), 32 with type-2 (nephrosclerosis) and 123 with type-3 (glomerulonephritis) CKD. The HD serum samples were collected from the Chiba Prefectural Sawara Hospital, and Port Square Kashiwado Clinic (Chiba, Japan). The HDs were individuals with no history of ischemic stroke or CKD, and no abnormalities upon a physical examination or brain magnetic resonance imaging. Those with autoimmune diseases were excluded from the study. The median (range) of age in years of the HDs, type-1 CKD, type-2 CKD and type-3 CKD subjects were 57 (44-76), 65 (38-93), 79 (54-90) and 63 (28-89), respectively. The subject information of the CKD cohort is summarized in Table SII. Each sample was centrifuged at 3,000 x g for 10 min at 4˚C, and the supernatant was stored at -80˚C until use. Repeated thawing and freezing were avoided.

The present study was conducted in accordance with the principles of the 1913 revision of the Declaration of Helsinki and with the approval of the Ethical Review Committee of Chiba University, Graduate School of Medicine and cooperative hospitals (approval no. 2018-320). The research on recombinant DNA was conducted with the permission of the Graduate School of Medicine, Chiba University, and following Japanese regulations. Each participant provided written informed consent to participation and publication.

### ProtoArray^®^ screening

The initial screening was performed using ProtoArray^®^ Human Protein Microarrays v.4.0 (Thermo Fisher Scientific, Inc.), which are loaded with 9,480 proteins, as previously described (18). A total of 20 serum samples (10 each from HDs and patients with atherosclerosis) were used to select antigenic proteins specifically recognized by serum immunoglobin G (IgG) antibodies. The results were analyzed using Prospector (Thermo Fisher Scientific, Inc.) software based on M-statistics. When comparing the two groups, a positive cutoff value corresponds to relative fluorescence units above the M-statistic signal threshold established for each antigenic protein. The percentage of the samples in each group that exhibited an immune response above the cutoff value was determined and the P-value for the significance of the difference between the two groups was calculated, as previously described (19).

### Expression and purification of glutathione-S-transferase-tagged antigenic proteins

The expression plasmid of the TSTD2 protein tagged with glutathione-S-transferase (GST) was constructed by recombining the cDNA sequence into a pGEX-4T-1 (Cytiva) plasmid vector. The competent *Escherichia coli* (*E. coli*) BL-21 cells was obtained from Nippon Gene, Co. Ltd. pGEX-4T-1-TSTD2 (0.2 µg in 1 µl) was mixed with the BL-21 cells (5x10^8^ cells in 20 µl), incubated for 5 min at 0˚C, incubated for 45 sec at 42˚C, and plated on agar-Luria broth plate containing 50 µg/ml ampicillin according to the instructions of Nippon Gene, Co. Ltd. The transformed cells were cultured in 200 ml Luria broth and treated with 0.1 mM isopropyl β-D-1-thiogalactopyranoside (IPTG) reagent for 3 h. The cells were then harvested, washed with phosphate-buffered saline (PBS), and sonicated in BugBuster Master Mix (Merck KGaA). The cell lysates were centrifuged at 13,000 x g for 10 min at 4˚C, and the precipitates containing recombinant proteins were dissolved in 8 M urea in TED buffer [50 mM Tris-HCl (pH 8.0), 1 mM ethylenediaminetetraacetic acid (EDTA) and 1 mM dithiothreitol]. The samples were then sequentially dialyzed in steps of 2 h each against 4 and 2 M urea in TED buffer. The samples were then dialyzed against TED buffer for 15 h to remove the urea and centrifuged at 10,000 x g for 30 min at 4˚C. The GST-fused recombinant protein recovered in the supernatant fraction was directly purified by affinity chromatography using glutathione-Sepharose (Cytiva) according to the manufacturer's instructions. The purified protein was filtered using Amicon Ultra-15 (Merck) centrifugal filter equipment to concentrate it (9,20-22). The concentration of the purified protein was quantified by Bradford method using Bio-Rad Protein assay (#5000001JA, Bio-Rad Laboratories, Inc.). The proteins were subjected to sodium dodecyl sulfate-polyacrylamide (11%) gel electrophoresis (SDS-PAGE) followed by staining with 0.05% Coomassie Brilliant Blue (Nakalai Tesque, Inc.) in 10% methanol (FUJIFILM Wako Pure Chemical Corporation) and 50% methanol (FUJIFILM Wako Pure Chemical Corporation) for 1 h at 25˚C and destaining in 7.5% acetic acid and 5% methanol for longer than 16 h at 25˚C. The purity was calculated using Personal Density Scanning Imager (Molecular Dynamics, Inc.).

### Western blot analysis

The GST and GST fusion proteins (0.3 µg) were separated by SDS-PAGE (11% polyacrylamide) and transferred to nitrocellulose membranes (cat. no. 1620112, Advantec Toyo Kaisha, Ltd.). The membranes were blocked with blocking solution [0.5% skim milk powder in a buffer consisting of 20 mM Tris-HCl (pH 7.6), 137 mM NaCl, and 0.1% Tween-20], and subjected to specific primary antibodies, i.e., 1:5,000-diluted antibodies against GST (cat. no. 600-101-200, Rockland Immunochemicals, Inc.) or 1:1,000-diluted sera from subjects. Following incubation with 1:30,000-diluted horseradish peroxidase (HRP)-conjugated secondary antibodies [donkey anti-goat (cat. no. sc-2056), or anti-human IgG (cat. no. sc-2453); both from Santa Cruz Biotechnology, Inc.], immunoreactivity was measured using Immobilon Western HRP Substrate (Merck KGaA) and LuminoGraph II (ATTO Co, Ltd.). The results were detected as previously described (21,23).

### Quantification of antibodies using amplified luminescent proximity homogenous assay-linked immunosorbent assay (AlphaLISA)

AlphaLISA was used for the quantitative measurement of serum antibodies to purified proteins. Subsequently, 2.5 µl serum diluted 1:100 in AlphaLISA buffer [25 mM hydroxyethyl piperazine ethane sulfonic acid (Thermo Fisher Scientific, Inc.), pH 7.4, 0.1% casein (Merck KGaA), 0.5% Triton X-100 (FUJIFILM Wako Pure Chemical Corporation), 1 mg/ml dextran-500 (Merck KGaA) and 0.05% Proclin-300 (Merck KGaA)] and 2.5 µl GST or GST-fused TSTD2 proteins (10 µg/ml) was placed in 384-well microtiter plates (white opaque OptiPlate, PerkinElmer, Inc.) and used for the experiments. The reaction mixture was incubated at room temperature for 6-8 h, after which anti-human IgG-conjugated acceptor beads (2.5 µl, 40 µg/ml; PerkinElmer, Inc.) and glutathione-conjugated donor beads (2.5 µl, 40 µg/ml; PerkinElmer, Inc.) were added. The mixture was incubated for 7-21 days at room temperature in the dark. Chemiluminescence was read on an EnSpire Alpha (PerkinElmer, Inc.) microplate reader (PerkinElmer, Inc.), as previously described (13,22).

### Statistical analyses

The Dunn's multiple comparison test following a Kruskal-Wallis test was used to analyze continuous variables using JMP Pro 14.2.0 software (SAS Institute Inc.). Correlations between TSTD2 antibody levels and each clinical and demographic parameter were evaluated using Spearman's correlation analysis. The cutoff TSTD2 antibody level for predicting ischemic stroke was assessed to maximize the sum of the sensitivity and specificity rates using a receiver operating characteristic (ROC) curve analysis. Clinical and demographic factors, including sex, age, smoking and alcohol drinking habits, and the presence of medical conditions such as hypertension, diabetes, cardiovascular disease, hyperlipidemia and obesity [based on body mass index (BMI)] were examined in relation to the serum TSTD2 antibody levels using a Mann-Whitney U test. Spearman's correlation analyses, ROC analysis and analysis using the Mann-Whitney U test were performed using GraphPad Prism 5 software (GraphPad, Inc.). All the tests were two-tailed, and a P-value <0.05 was considered to indicate a statistically significant difference.

## Results

### Identification of the TSTD2 antibody using ProtoArray screening

**Figure 1:**
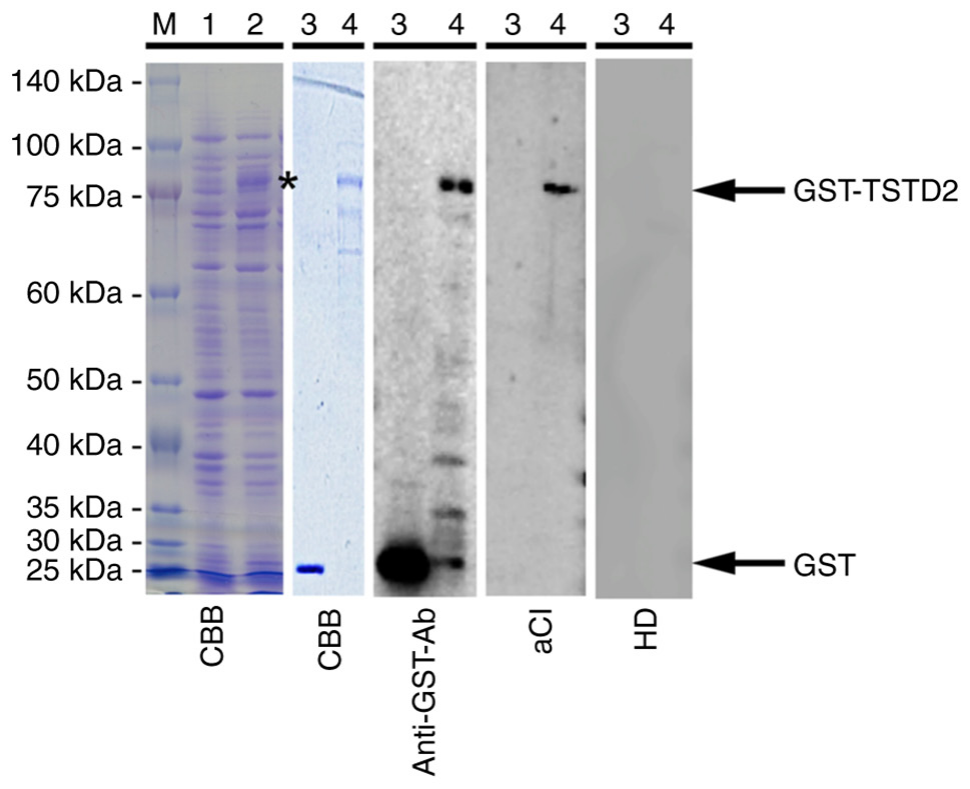
Western blot analysis. *Escherichia coli* BL-21 cells containing the pGEX-4T-1-TSTD2 were treated without (lane 1) or with 0.1 mM isopropyl β-D-1-thiogalactopyranoside (lane 2) for 3 h at 42˚C in Luria broth. The whole cell extract was electrophoresed with sodium dodecyl sulfate-polyacrylamide (11%) gels followed by staining with CBB. The asterisk represents GST-tagged full-length TSTD2 (GST-TSTD2) protein in the whole cell extract. Affinity-purified GST (lane 3) and GST-TSTD2 proteins (lane 4) were electrophoresed similarly and then stained with CBB or used for western blotting with anti-GST antibodies or the serum antibodies of a patient with aCI and those of a HD. Electrophoresed molecular weight markers are also shown in lane M, with the sizes shown on the left. CBB, Coomassie Brilliant Blue; GST, glutathione-S-transferase; aCI, acute cerebral infarction; HD, healthy donor.

The screening of ProtoArray^®^ loaded with 9,480 proteins identified TSTD2 antibodies (accession no. NM_139246.3) in eight of the 10 serum samples from patients with atherosclerosis and in two of the 10 serum samples from the HDs. The expression of GST-fused TSTD2 protein was successively induced in *E. coli* containing pGEX-4T-1-TSTD2 following treatment with IPTG (Fig. 1, lanes 1 and 2 of the CBB blot). Affinity-purified GST and GST-TSTD2 proteins were also subjected to SDS-PAGE followed by staining with Coomassie Brilliant Blue (Fig. 1, lanes 3 and 4 of the CBB blot). The purity of GST-TSTD2 protein was ~80%.

### Elevated TSTD2 antibody levels in patients with AIS

**Figure 2:**
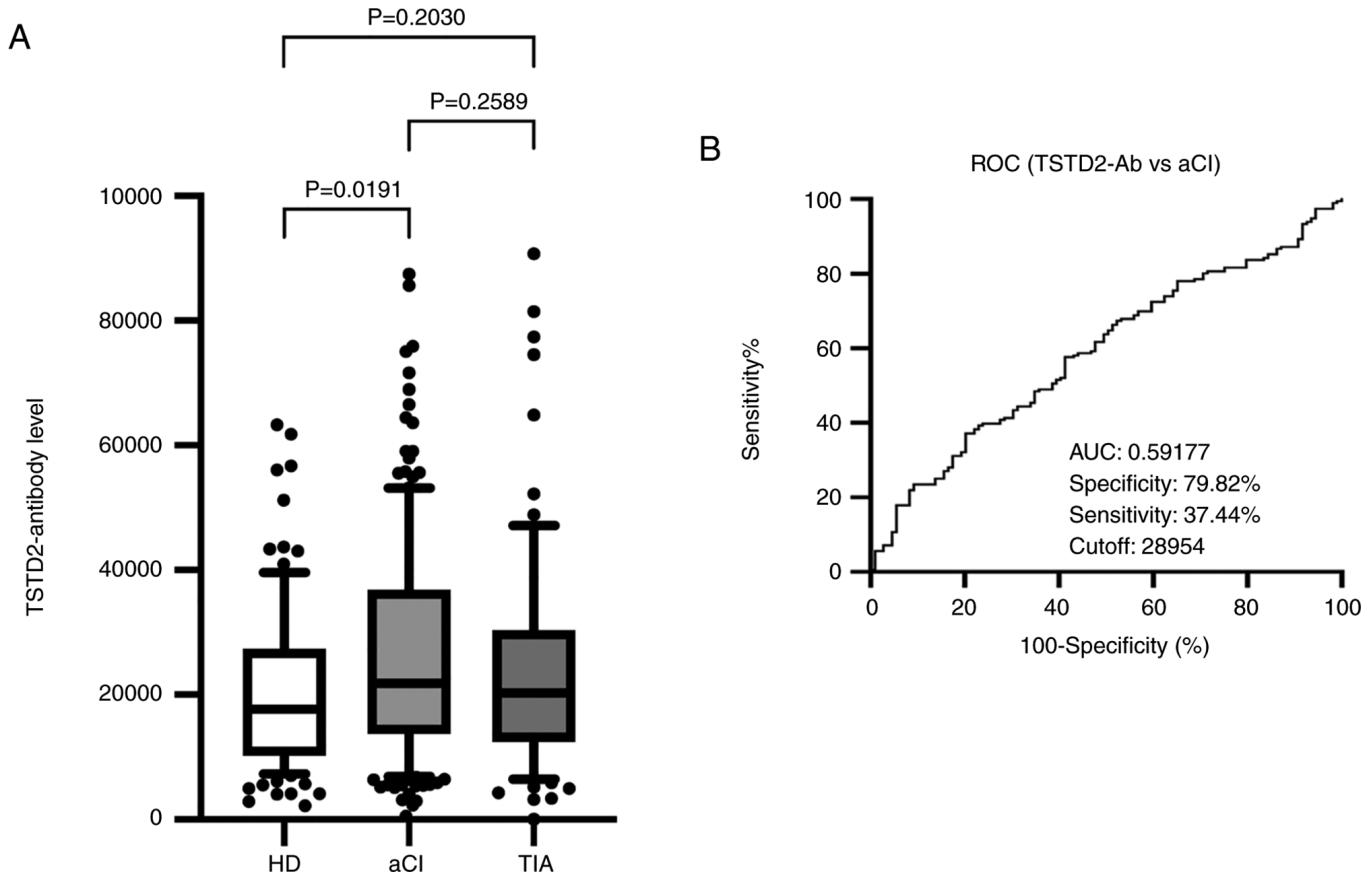
Comparison of the serum levels of TSTD2 antibodies in HDs, and in patients with aCI and TIA. (A) The figure illustrates the levels of serum TSTD2-Abs examined using amplified luminescence proximity homogeneous assay (Alpha)-linked immunosorbent assay. Antibody levels are represented by Alpha photon counts and shown in a box-whisker plot. The horizontal lines represent medians, and the boxes represent the 25 and 75th percentiles. The whiskers represent the 10 and 90th percentiles, and the dots represent outliers. P-values were calculated using the Kruskal-Wallis test. A ROC curve analysis was performed to assess the ability of serum TSTD2-Abs to detect aCI. (B) The numbers in the graph are the AUC, specificity, sensitivity and cutoff values for the marker levels. TSTD2, thiosulfate sulfurtransferase-like domain-containing 2; TSTD2-Ab, TSTD2 antibody; HD, healthy donor; aCI, acute cerebral infarction; TIA, transient ischemic attack; ROC, receiver operating characteristic; AUC, area under the curve.

To investigate the association between TSTD2 autoantibodies and AIS, serum TSTD2 antibody levels were examined in the HD, aCI and TIA groups using AlphaLISA. Compared with the HD group, the aCI group exhibited significantly higher TSTD2 antibody levels (P=0.0191); however, no significant difference was observed between the TIA and HD groups (P=0.2030), or between the TIA and aCI groups (P=0.2589) (Fig. 2A).

### Elevation of TSTD2 antibody levels in patients with CKD

**Figure 3:**
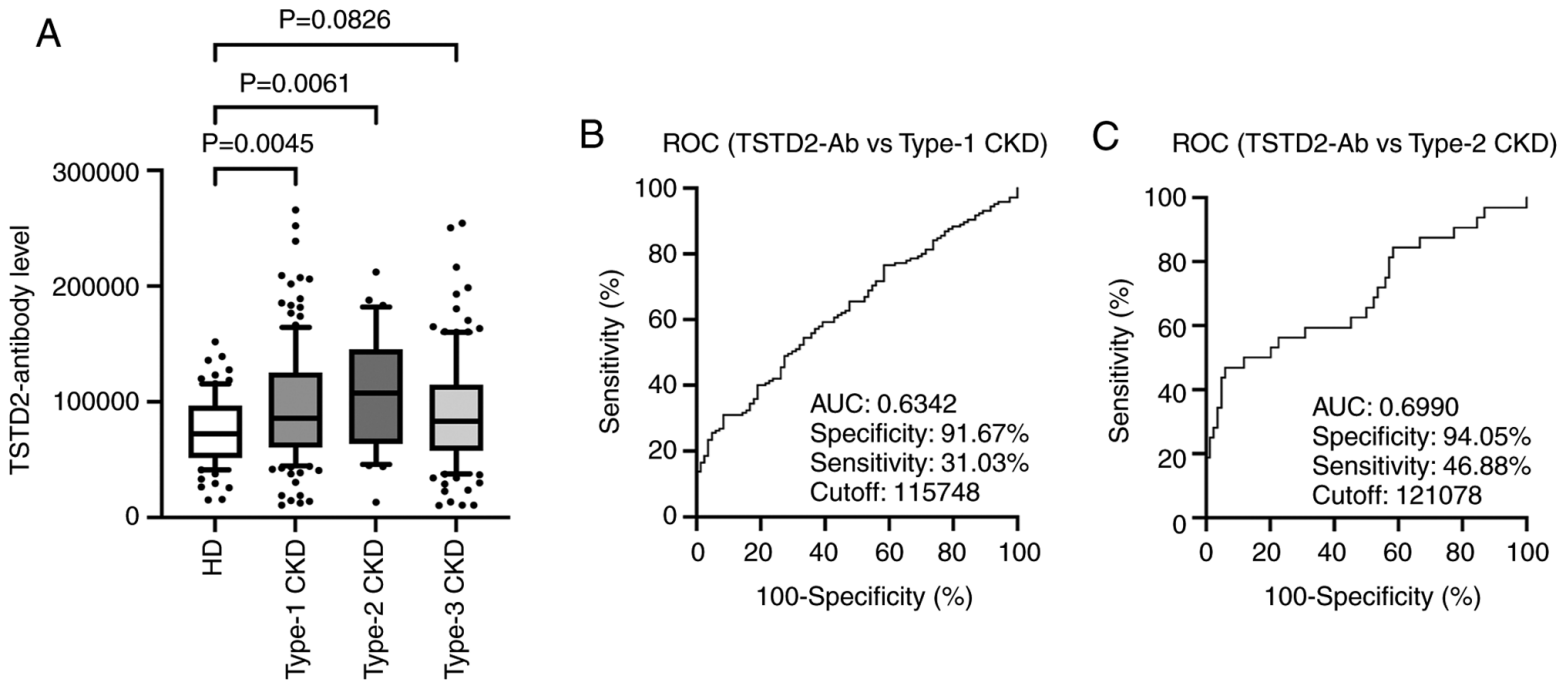
Comparison of serum TSTD2 antibody levels in HDs, and patients with type-1, -2 and -3 CKD. (A) Serum TSTD2 antibody levels were quantified using amplified luminescence proximity homogeneous assay-linked immunosorbent assay and compared between the HD group and the type-1 (diabetic kidney disease), type-2 (nephrosclerosis) and type-3 (glomerulonephritis) CKD subgroups. TSTD2 antibody levels are depicted in a box-whisker plot. (B and C) The TSTD2 antibody levels were analyzed using a ROC curve to compare type-1 and type-2 CKDs. The numbers in the graphs indicate the AUC, specificity, sensitivity and cutoff values. TSTD2, thiosulfate sulfurtransferase-like domain-containing 2; CKD, chronic kidney disease; ROC, receiver operating characteristic; AUC, area under the curve.

The present study then examined antibody levels in the sera of patients with CKD. This group was divided into three subgroups according to CKD type as follows: Type-1 (diabetic kidney disease), type-2 (nephrosclerosis) and type-3 (glomerulonephritis). The type-1 and type-2 CKD subgroups had significantly higher serum TSTD2-Ab levels than the HDs. The type-3 CKD subgroup exhibited higher levels than the HD group, although the difference was not significant (P=0.0826) (Fig. 3A).

### ROC analysis of TSTD2 antibody levels in patients with aCI and type-1 and type-2 CKDs

A ROC analysis of the TSTD2 antibody levels in aCI and type-1 and type-2 CKDs was also performed. The cutoff values for these levels in aCI and type-1 and type-2 CKDs were 28,954 (sensitivity, 37.4%; specificity, 79.8%), 11,5748 (sensitivity, 31.03%; specificity, 91.67%) and 12,1078 (sensitivity, 46.88%; specificity, 94.05%), respectively (Figs. 2B and 3B and C). The area under the curve (AUC) values were 0.592, 0.6342 and 0.6990 for aCI and type-1 and type-2 CKDs, respectively (Figs. 2B, 3B and C). The AUC value for type-2 CKD was higher than that for type-1 CKD, although the sample numbers examined diffed.

### Analysis of correlations between TSTD2 antibody levels and clinical and demographic parameters in the stroke cohort

Spearman's correlation analyses to identify the correlations between the TSTD2 antibody levels, and the clinical and demographic variables in the Sawara stroke cohort. The patient information is presented in Table SI, which included hematological examination results, smoking status and alcohol consumption status, age, sex, height, weight, BMI and intima-media thickness (IMT) of the carotid artery. Positive correlations were found between the TSTD2 antibody levels and maximum IMT (Rho=0.1703, P=0.0028), C-reactive protein levels (Rho=0.1395, P=0.0221), blood sugar (Rho=0.1542, P=0.039), smoking duration (Rho=0.1938, P=0.0002) and alcohol consumption frequency (Rho=0.1174, P=0.0241). Negative correlations were found between the TSTD2 antibody levels and cholinesterase levels (Rho=-0.1960, P=0.0009), albumin levels (Rho=-0.1727, P=0.0007) and total cholesterol (Rho=-0.1567, P=0.0046) (Table I).

**Figure 4:**
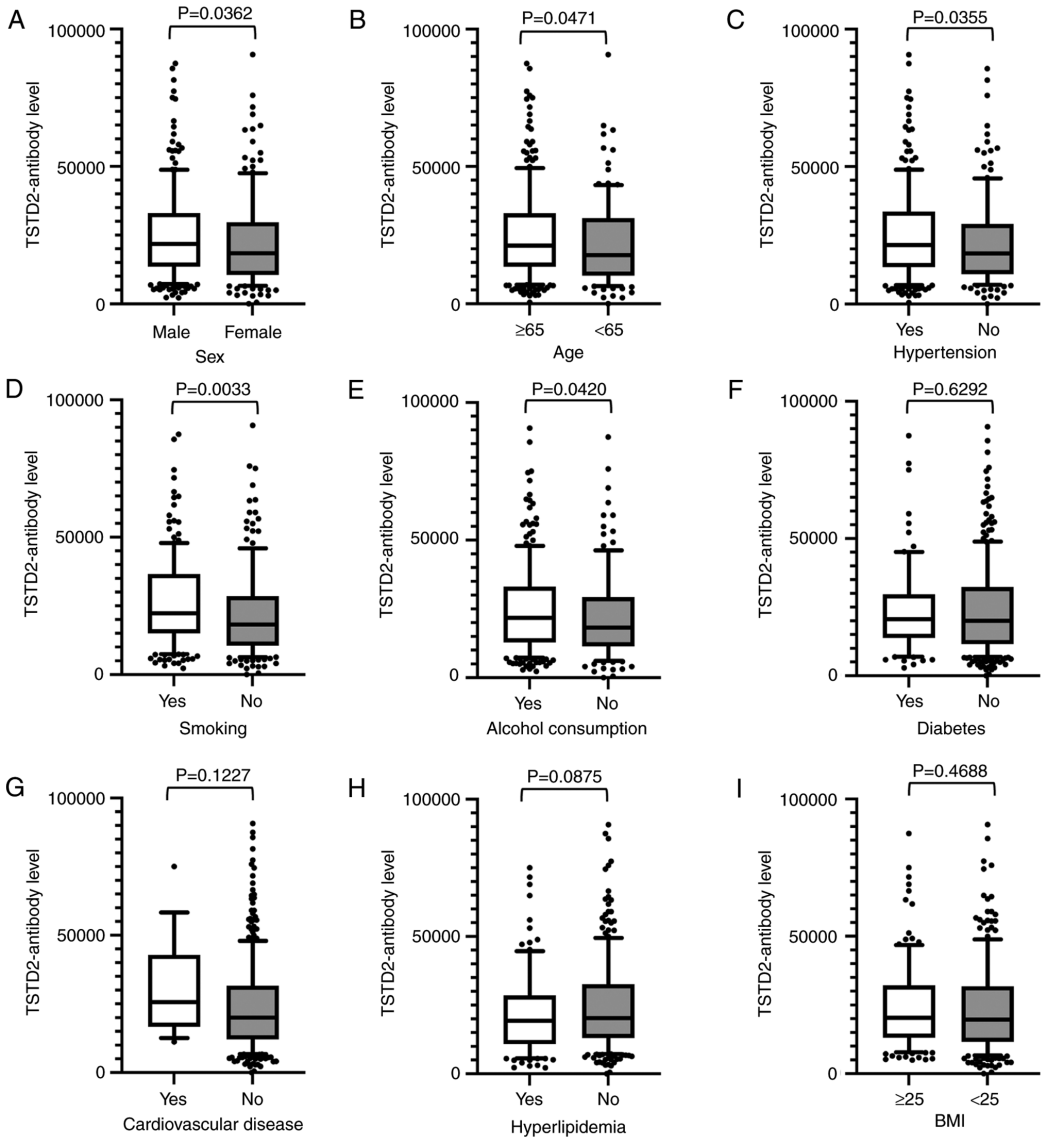
Associations between TSTD2 antibody levels and clinical and demographic variables in the serum of acute cerebral infarction patients. The associations between TSTD2 antibody levels and (A) sex, (B) age, (C) hypertension, (D) smoking status, (E) alcohol consumption, (F) diabetes, (G) cardiovascular disease, (H) hyperlipidemia, and (I) BMI were examined in the acute cerebral infarction cohort. The TSTD2 antibody levels obtained using amplified luminescence proximity homogeneous assay-linked immunosorbent assay are shown in box-whisker plots. The P-values were calculated using Mann-Whitney U tests. TSTD2, thiosulfate sulfurtransferase-like domain-containing 2; BMI, body mass index.

Antibody levels were compared between two groups using the Mann-Whitney U test using the cutoff values determined by ROC analysis, and were found to be significantly higher in males than in females (P=0.036), those who were ≥65 years of age compared with those <65 of age (P=0.047), in those with hypertension compared with those without this condition (P=0.036), in smokers compared with non-smokers (P=0.003), and in those who consumed alcohol compared with those who did not (P=0.042) (Fig. 4).

### Analysis of correlations between TSTD2 antibody levels and clinical and demographic parameters in the CKD cohort

Subsequently, Spearman's correlation analysis was performed to identify the correlations between the serum TSTD2 antibody levels and the clinical features of patients in the Kumamoto CKD cohort. The patient information is presented in Table SII. Positive correlations were found between TSTD2 antibody levels and plaque scores (Rho=0.1608, P=0.0056), maximum IMT (Rho=0.1266, P=0.0295), cardio-ankle vascular index (CAVI) of the right side (Rho=0.1430, P=0.0165) and the left side (Rho=0.1472, P=0.0132), parathyroid hormone (Rho=0.1539, P=0.0076), aspartate aminotransferase (Rho=0.1654, P=0.0041), lactate dehydrogenase (Rho=0.1749, P=0.0024), and C-reactive protein (Rho=0.1638, P=0.0044) levels. A negative correlation was found between TSTD2 antibody levels and BMI (Rho=-0.1509, P=0.0089) (Table II).

### The presence of autoantibodies against purified protein in the sera of aCI patients

Western blot analysis was performed to determine the presence of anti-TSTD2 antibodies in the serum samples. Both GST and GST-TSTD2 protein reacted with commercial anti-GST antibodies whereas GST-TSTD2, but not GST was recognized by antibodies in the sera of patients with aCI (Fig. 1, lanes 3 and 4 of the anti-GST-Ab, aCI, and HD blots). HD serum contained no TSTD2 antibodies.

## Discussion

Atherosclerosis is one of the major causes of ischemic stroke (24) and the primary cause of CKD (5). Autoantibodies have been found to develop alongside atherosclerosis (13,14). Some of these autoantibodies may have causal or suppressive effects on disease development. For example, anti-GRP78 autoantibodies accelerate the development of atherosclerotic lesions (25). Therefore, the present study performed ProtoArray^®^ screening and identified a novel autoantibody marker and selected the TSTD2 autoantibody as a candidate marker for atherosclerosis. AlphaLISA was then used to quantify anti-TSTD2 antibodies in patient sera, and a significant increase in the antibody levels was found in samples from patients with aCI and type-1 and type-2 CKD, as compared with those from the HDs (Figs. 2A and 3A). Western blot analysis was performed and this confirmed the presence of TSTD2 antibodies in aCI sera and their absence in HD sera (Fig. 1).

Subsequently, the correlation between clinical factors and anti-TSTD2 antibody levels was examined in the aCI cohort. A significant elevation in antibody levels was observed in males, and in those with hypertension, who were older, and with a smoking history and a history of alcohol consumption (Fig. 4), all of which are risk factors for atherosclerosis (26-28). These findings were consistent with the results of the Spearman's correlation analyses, which revealed a positive correlation between the serum TSTD2 antibody levels and maximum IMT and smoking (Table I). Maximum IMT, plaque score and CAVI (left and right) were also associated with serum TSTD2 antibody levels in the CKD cohort (Table II). IMT and plaque scores are widely accepted as indicators of atherosclerosis (29,30). Thus, the TSTD2 antibody may predominantly reflect the development of atherosclerosis, and may thus be associated with the presence of aCI and CKD.

Spearman's correlation analysis revealed that blood sugar, a typical diabetes mellitus (DM) marker, was related to the serum TSTD2 antibody levels (Table I). This correlation did not remain significant when the presence of DM was accounted for (Fig. 4F). The AUC of type-2 CKD (nephrosclerosis) was higher than that for type-1 CKD (diabetic kidney disease) (Fig. 3B and C). Consequently, it can be concluded that the serum TSTD2-Abs levels do not directly reflect DM, but may be related to DM-induced atherosclerotic disorders, including CKD. This is in contrast to anti-SH3BP5 antibody marker, which is mainly related to DM and, along with that, with type-1 CKD (31). Anti-DIDO1 antibody marker has been found to be equally associated with all three types of CKD, but has shown no association with DM (14).

TSTD2 is a thiosulfate sulfurtransferase (31). Although the function of TSTD2 has not yet been fully elucidated, its expression in the soleus feed artery can be upregulated by endurance exercise training (32). TSTD2 can function in the downstream region of the (pro)renin receptor signaling pathway (33). The renin-angiotensin system plays a key regulatory role in hypertension (34). This is consistent with the finding of the present study that the TSTD2 antibody levels were strongly associated with hypertension (Fig. 4C). If TSTD2 plays a causal role in the regulation of hypertension, it may be used as a therapeutic target to prevent the development of atherosclerosis. Among the other correlated factors in Fig. 4, the habit of smoking is one of the major risk factors for the development of atherosclerosis (35). Age may be indirectly associated with atherosclerosis as a confounding factor. The male sex and alcohol consumption may also be associated with the smoking habit and indirectly with atherosclerosis.

Reactive oxygen species (ROS) are considered to contribute to vascular inflammation in atherosclerosis as a result of mediating various signaling pathways (36,37). As a result, there are increasing reports that the excessive production of ROS is implicated in the pathogenesis of acute and chronic cardiovascular diseases and renal dysfunction (38-40). TSTD2 is one of the TST-like domains and is known as rhodanese (41). It is a mitochondrial enzyme that catalyzes sulfur transfer through multiple molecular pathways and is known for its ability to reduce the antioxidants glutathione and thioredoxin, detoxify ROS and regulate cellular homeostasis (42,43). Although, to the best of our knowledge, there have been no reports to date demonstrating a direct association between TSTD2 function and ROS, TST has antioxidant effects, and smoking, alcohol consumption, and hyperglycemia have the potential to cause vascular endothelial damage due to oxidative stress and the production of free radicals. It was hypothesized speculated that the levels of TSTD2, which is considered to contribute to the detoxification of ROS, are increased in blood, resulting in an elevation in the levels of anti-TSTD2 antibodies.

The early stages of atherosclerosis are usually accompanied by elevated autoantibody levels (44). Autoantibodies are stable and easy to detect in patients' sera (45). Hence, atherosclerosis-induced autoantibodies can be used for the early diagnosis of atherosclerosis-related diseases. The present study identified the TSTD2 antibody as a novel biomarker that may be used for the detection of atherosclerosis-related aCI and CKD. However, further research is required to evaluate the sensitivity and specificity using validation cohorts, including atherosclerosis-related diseases, as well as disease controls.

## Supplementary Material

Subject information of the Sawara stroke cohort.

Subject information of the Kumamoto CKD cohort.

## Figures and Tables

**Table I:** Spearman's correlation analysis for the correlation between the serum TSTD2 antibody levels and clinical features of patients with aCI.

Variables	Spearman's rank correlation coefficient (Rho)	P-value
Age, years	0.0954	0.0624
BMI	0.0193	0.7075
Maximum IMT	0.1703	**0.0028**
AST	0.0054	0.9161
ALT	-0.0428	0.4051
ALP	0.0647	0.228
LDH	0.086	0.0986
tBil	-0.0226	0.6642
CHE	-0.1960	**0.0009**
γ-GTP	0.0586	0.2677
TP	-0.0910	0.0797
ALB	-0.1727	**0.0007**
BUN	0.034	0.5082
Creatinine	-0.0079	0.8783
eGFR	-0.0124	0.8154
UA	0.069	0.2558
AMY	-0.0764	0.2537
T-CHO	-0.1567	0.0046
HDL-C	-0.0673	0.3106
TG	-0.1109	0.0814
Na	-0.0139	0.7877
K	-0.0994	0.054
Cl	-0.0989	0.055
CRP	0.1395	**0.0221**
WBC	0.0905	0.0783
RBC	-0.0673	0.1912
HGB	-0.0564	0.2737
HCT	-0.0626	0.2243
PLT	-0.0481	0.3503
BS	0.1542	**0.0039**
HbA1c	-0.0250	0.6695
Smoking duration (years)	0.1938	**0.0002**
Frequency of alcohol consumption (times/week)	0.1174	**0.0241**

A correlation analysis was performed to identify the correlation between TSTD2 antibody levels and the clinical features of patients with ischemic stroke. Correlation coefficients (Rho) and P-values were calculated using Spearman's correlation analysis. Significant correlations (P<0.05) are marked in bold font. aCI, acute cerebral infarction; TSTD2, thiosulfate sulfurtransferase-like domain-containing 2; BMI, body mass index; IMT, intima-media thickness; AST, aspartate aminotransferase; ALT, alanine aminotransferase; ALP, alkaline phosphatase; LDH, lactate dehydrogenase; tBil, total bilirubin; CHE, choline esterase; γ-GTP, γ-glutamyl transpeptidase; TP, total protein; ALB, albumin; BUN, blood urea nitrogen; eGFR, estimated glomerular filtration rate; UA, uric acid; AMY, amylase; T-CHO, total cholesterol; HDL-C, high-density lipoprotein cholesterol; TG, triglyceride; CRP, C-reactive protein; WBC, white blood cell count; RBC, red blood cell count; HCT, hematocrit; PLT, platelet count; BS, blood sugar; HbA1c, hemoglobin A1c.

**Table II:** Spearman's correlation analysis of the correlation between the serum TSTD2 antibody levels and clinical features of patients with CKD.

Variables	Spearman's rank correlation coefficient (Rho)	P-value
Age, years	0.0545	0.3468
Height	-0.0044	0.9401
Weight	-0.1152	0.0461
BMI	-0.1509	**0.0089**
Plaque score	0.1608	**0.0056**
Maximum IMT	0.1266	**0.0295**
ABI (right)	-0.0122	0.8353
ABI (left)	-0.0124	0.8319
CAVI (right)	0.1430	**0.0165**
CAVI (left)	0.1472	**0.0132**
HbA1c	-0.0077	0.9260
PTH	0.1539	**0.0076**
Fe	-0.0984	0.0889
Ferritin	0.1110	0.0547
TSAT ratio	-0.0615	0.2882
Kt/V	-0.0874	0.1308
RBC	-0.0680	0.2406
PLT	-0.0743	0.1994
TP	-0.0568	0.3272
ALB	-0.0758	0.1907
UA	-0.0153	0.7912
Na	0.1010	0.0807
K	-0.0112	0.8462
Cl	0.0522	0.3676
Ca	0.0130	0.8223
IP	-0.0017	0.9770
Mg	0.0544	0.3476
AST	0.1654	**0.0041**
ALT	0.0975	0.0920
LDH	0.1749	**0.0024**
γ-GTP	0.0895	0.1219
AP	0.0557	0.3366
tBil	-0.0105	0.8559
AMY	-0.0448	0.4394
Creatinin	-0.0354	0.5415
T-CHO	-0.0300	0.6047
HDL-C	-0.0741	0.2004
LDL-C	-0.0058	0.9197
TG	0.0383	0.5086
CRP	0.1638	**0.0044**

A correlation analysis was performed to identify the correlation between TSTD2 antibody levels and the clinical features in patients with CKD. Correlation coefficient (Rho) and P-values were calculated using Spearman's correlation analysis. Significant correlations (P<0.05) are marked in bold font. TSTD2, thiosulfate sulfurtransferase-like domain-containing 2; CKD, chronic kidney disease; BMI, body mass index; maximum IMT, maximum intima-media thickness; ABI, ankle brachial pressure index; CAVI, cardio-ankle vascular index; HbA1c, glycated hemoglobin; W-PTH, whole parathyroid hormone; ARB, angiotensin II receptor blocker; ACE, angiotensin converting enzyme; PTA, prothrombin; TSAT ratio, transferrin saturation ratio; Kt/V, standardized urea clearance; RBC, red blood cell number; PLT, platelet number; TP, total protein; ALB, albumin; UA, uric acid; HGB, hemoglobin; HCT, hematocrit; UN, urea nitrogen; CRE, creatinine; IP, inorganic phosphate; AST, aspartate aminotransferase; ALT, alanine amino transferase; LDH, lactate dehydrogenase; γ-GTP, γ-glutamyl transpeptidase; AP, alkaline phosphatase; tBil, total bilirubin; AMY, amylase; T-CHO, total cholesterol; HDL-C, high-density lipoprotein cholesterol; LDL-C, low-density lipoprotein cholesterol; TG, triglyceride; and CRP, C-reactive protein.

## Data Availability

All results of the ProtoArray^®^ Human Protein Microarrays are available in the Figshre database (https://doi.org/10.6084/m9.figshare.21510009.v1).
