# Supplementary material for: Serum anti‑TSTD2 antibody as a biomarker for atherosclerosis‑induced ischemic stroke and chronic kidney disease
Source: Med Int (Lond). 2022 Dec 21;3(1):4. doi: 10.3892/mi.2022.64 (PMC9829233; doi:10.3892/mi.2022.64)
Supplement: Subject information of the Sawara stroke cohort. [file Supplementary_Data.pdf]

Table SI. Subject information of the Sawara stroke cohort.

Parameter	HD	aCI	TIA
Total sample no.	109	196	79
Sex (male/female)	62/47	122/74	46/33
Age, years			
Average ± SD	59.8±7.9	75.1±7.3	70.7±12.8
Median (range)	60 (45-90)	77 (58-85)	73 (26-90)
Diabetes, no. (%)	9 (8.3%)	53 (27.0%)	19 (24.1%)
Hypertension, no. (%)	34 (31.2%)	153 (78.1%)	53 (67.1%)
Cardiovascular disease, no. (%)	1 (0.9%)	10 (5.1%)	4 (5.1%)
Hyperlipidemia, no. (%)	31 (28.4%)	56 (28.6%)	30 (38.0%)
Smoking habits, no. (%)	47 (43.1%)	107 (54.6%)	29 (36.7%)
BMI, average ± SD	23.4.3	23.0±3.5	23.6±3.7
Maximum IMT (mm), average ± SD	1.7±0.8	3.0±1.6	2.5±1.3
AST (U/l), average ± SD	27.9±35.0	23.4±9.2	27.7±31.9
ALT (U/l), average ± SD	24.1±21.5	19.0±10.2	26.8±39.1
ALP (U/l), average ± SD	239±119	241±76	242±73
LDH (U/l), average ± SD	189±39	200±48	194±45
tBil (mg/dl), average ± SD	0.9±0.4	0.8±0.4	0.7±0.3
CHE (U/l), average ± SD	345±86	304±81	313±78
γ-GTP (U/l), average ± SD	37.9±50.7	31.5±27.9	37.6±45.1

TP (g/dl), average ± SD	7.2±0.5	7.3±1.0	7.1±0.5
ALB (g/dl), average ± SD	4.4±0.4	4.2±0.5	4.2±0.5
BUN (mg/dl), average ± SD	13.7±3.2	16.3±9.0	15.5±6.1
Creatinine (mg/dl), average ± SD	0.7±0.2	0.9±1.0	0.8±0.3
eGFR (mL/min/1.73m^2), average ± SD	79.0±20.4	71.1±22.3	70.7±19.8
UA (mg/dl), average ± SD	5.1±1.4	5.6±4.5	5.4±1.3
AMY (U/l), average ± SD	178±109	181±72	135±63
T-CHO (mg/dl), average ± SD	201±37	196±37	204±43
HDL-C (mg/dl), average ± SD	58.9±16.9	54.4±21.9	52.5±14.3
TG (mg/dl), average ± SD	117±84	121±69	127±77
Na (mEq/l), average ± SD	140±2	140±2	140±2
K (mq/l), average ± SD	4.1±0.3	4.9±9.8	4.1±0.4
Cl (mEq/l), average ± SD	104±3	104±8	105±3
CRP (mg/dl), average ± SD	0.4±1.5	1.3±4.0	0.3±0.5
WBC (x10^3/µl), average ± SD	6.2±2.0	7.7±8.5	6.5±1.7
RBC (x10^6/µl), average ± SD	4.6±0.5	4.5±0.6	4.4±0.6
HGB (g/dl), average ± SD	14.1±1.9	13.9±2.0	13.4± .9
HCT (%), average ± SD	41.1±4.8	40.8±5.3	39.8±5.1
PLT (x10^3/µl), average ± SD	219±49	219±63	225±154

BS (mg/dl), average ± SD	114±36	138±57	125±48
HbA1c (%), average ± SD	5.6±0.7	5.8±1.2	5.8±0.9
Smoking period (year), average ± SD	12.5±16.6	18.6±22.0	16.1±19.5
Alcohol drinking frequency (time/w), average ± SD	2.6±3.2	2.0±3.0	2.4±3.0

HD, healthy donor; aCI, acute cerebral infarction; TIA, transient ischemic attack; BMI, body mass index; IMT, intima-media thickness; AST, aspartate aminotransferase; ALT, alanine aminotransferase; ALP, alkaline phosphatase; LDH, lactate dehydrogenase; tBil, total bilirubin; CHE, choline esterase; γ-GTP, gamma-glutamyl transpeptidase; TP, total protein; ALB, albumin; BUN, blood urea nitrogen; eGFR, estimated glomerular filtration rate; UA, uric acid; AMY, amylase; T-CHO, total cholesterol; HDL-C, high-density lipoprotein cholesterol; TG, triglyceride; CRP, C-reactive protein; WBC, white blood cell count; RBC, red blood cell count; HCT, hematocrit; PLT, platelet count; BS, blood sugar; HbA1c, hemoglobin A1c.

Table SII. Subject information of the Kumamoto CKD cohort.

Parameter	Type-1 CKD	Type-2 CKD	Type-3 CKD
Total sample no.	145	32	123
Sex, male/female	106/39	21/11	70/53
Age, years			
Average ± SD	66.0±10.4	76.0±9.8	62.0±11.7
Median (range)	65 (38 - 93)	79 (54 - 90)	63 (28 - 89)
Height (cm), average ± SD	161.1±9.1	156.1±9.8	159.5±9.0
Weight (kg), average ± SD	59.2±12.2	53.9±11.5	53.4±11.2
BMI, average ± SD	22.8±3.6	22.0±3.1	20.8±3.1
Plaque score, average ± SD	7.7±4.1	6.8±4.0	4.3±3.6
Maximum IMT (mm), average ± SD	2.2±0.8	2.1±0.7	1.8±0.9
ABI (right), average ± SD	1.03±0.18	1.04±0.19	1.12±0.14
ABI (left), average ± SD	1.03±0.20	1.02±0.20	1.11±0.14
CAVI (right), average ± SD	9.8±1.8	9.3±2.3	9.0±1.7
CAVI (left), average ± SD	9.8±1.8	9.1±2.5	8.8±1.7
HbA1c (%), average ± SD	6.0±1.2	5.4±0.8	5.5±0.5
PTH (pg/ml), average ± SD	69.3±66.0	58.7±54.9	70.3±96.0
Fe (μg/dl), average ± SD	62.5±28.0	63.4±29.5	67.2±23.1
Ferritin (ng/ml), average ± SD	87.0±110.0	87.2±93.2	62.1±89.0
TSAT ratio, average ± SD	24.7±10.5	23.9±10.3	25.8±10.2
Kt/V, average ± SD	1.36±0.23	1.33±0.29	1.56±0.25

RBC ($x10^6$/μl), average ± SD	3.7±0.5	3.5±0.4	3.6±0.4
PLT ($x10^3$/μl), average ± SD	164±48	166±48	169±46
TP (g/dl), average ± SD	6.7±0.5	6.6±0.4	6.6±0.4
ALB (g/dl), average ± SD	3.8±0.3	3.9±0.3	3.9±0.3
UA (mg/dl), average ± SD	7.8±1.3	8.0±1.5	8.1±1.1
Na (mEq/l),average ± SD	137±3	139±2	139±2
K (mq/l), average ± SD	4.7±0.8	4.6±0.6	4.9±0.6
Cl (mEq/l), average ± SD	102±3	103±3	102±3
Ca (mEq/l), average ± SD	9.1±0.7	9.5±0.9	9.3±0.7
IP (mEq/l), average ± SD	5.6±1.3	5.2±1.3	5.4±1.1
Mg (mEq/l), average ± SD	2.7±0.4	2.7±0.4	2.8±0.4
AST (U/l), average ± SD	12.9±6.2	13.9±5.1	14.4±8.0
ALT (U/l), average ± SD	11.7±6.7	10.1±3.8	11.5±7.1
LDH (U/l), average ± SD	185±36	197±39	191±33
γ-GTP (U/l), average ± SD	32.4±49.6	17.8±6.4	29.4±30.4
ALP (U/l), average ± SD	244±102	206±63	242±109
tBil (mg/dl), average ± SD	0.4±0.1	0.4±0.1	0.3±0.1
AMY (U/l), average ± SD	103±40	123±50	143±56
Creatinine (mg/dl), average ± SD	10.3±2.4	9.8±1.9	11.9±2.3
T-CHO (mg/dl), average ± SD	146±30	152±20	163±33
HDL-C (mg/dl), average ± SD	44.8±14.3	41.4±11.3	51.7±14.6
LDL-C (mg/dl), average ± SD	73.8±22.5	81.8±15.2	85.0±26.6
TG (mg/dl), average ± SD	114±85	112±61	107±69

CRP (mg/dl), average ± SD	2.5±5.4	2.6±6.2	1.4±2.1

CKD, chronic kidney disease; BMI, body mass index; maximum IMT, maximum intima–media thickness; ABI, ankle brachial pressure index; CAVI, cardio-ankle vascular index; HbA1c, glycated hemoglobin; W-PTH , whole parathyroid hormone; ARB, angiotensin II receptor blocker; ACE, angiotensin converting enzyme; PTA, prothrombin; TSAT ratio, transferrin saturation ratio; Kt/V, standardized urea clearance; RBC, red blood cell number; PLT, platelet number; TP, total protein; ALB, albumin; UA, uric acid; HGB, hemoglobin; HCT, hematocrit; UN, urea nitrogen; CRE, creatinine; IP, inorganic phosphate; AST, aspartate aminotransferase; ALT, alanine amino transferase; LDH, lactate dehydrogenase; γ-GTP, γ-glutamyl transpeptidase; AP, alkaline phosphatase; tBil, total bilirubin; AMY, amylase; T-CHO, total cholesterol; HDL-C, high-density lipoprotein cholesterol; LDL-C, low-density lipoprotein cholesterol; TG, triglyceride; and CRP, C-reactive protein.
